# Neuroprotection in Rats Following Ischaemia-Reperfusion Injury by GLP-1 Analogues—Liraglutide and Semaglutide

**DOI:** 10.1007/s10557-019-06915-8

**Published:** 2019-11-13

**Authors:** Maryna V. Basalay, Sean M. Davidson, Derek M. Yellon

**Affiliations:** The Hatter Cardiovascular Institute, University College London, 67 Chenies Mews, London, WC1E 6HX UK

**Keywords:** Acute ischaemic stroke, Glucagon-like peptide-1, Ischaemia-reperfusion injury, Middle cerebral artery occlusion, Neuroprotection

## Abstract

**Purpose:**

A substantial number of ischaemic stroke patients who receive reperfusion therapy in the acute phase do not ever fully recover. This reveals the urgent need to develop new adjunctive neuroprotective treatment strategies alongside reperfusion therapy. Previous experimental studies demonstrated the potential of glucagon-like peptide-1 (GLP-1) to reduce acute ischaemic damage in the brain. Here, we examined the neuroprotective effects of two GLP-1 analogues, liraglutide and semaglutide.

**Methods:**

A non-diabetic rat model of acute ischaemic stroke involved 90, 120 or 180 min of middle cerebral artery occlusion (MCAO). Liraglutide or semaglutide was administered either i.v. at the onset of reperfusion or s.c. 5 min before the onset of reperfusion. Infarct size and functional status were evaluated after 24 h or 72 h of reperfusion.

**Results:**

Liraglutide, administered as a bolus at the onset of reperfusion, reduced infarct size by up to 90% and improved neuroscore at 24 h in a dose-dependent manner, following 90-min, but not 120-min or 180-min ischaemia. Semaglutide and liraglutide administered s.c. reduced infarct size by 63% and 48%, respectively, and improved neuroscore at 72 h following 90-min MCAO. Neuroprotection by semaglutide was abolished by GLP1-R antagonist exendin(9-39).

**Conclusion:**

Infarct-limiting and functional neuroprotective effects of liraglutide are dose-dependent. Neuroprotection by semaglutide is at least as strong as by liraglutide and is mediated by GLP-1Rs.

**Electronic supplementary material:**

The online version of this article (10.1007/s10557-019-06915-8) contains supplementary material, which is available to authorized users.

## Introduction

At present, ischaemic stroke remains one of the most costly and devastating clinical syndromes in the world [1]. Recently, endovascular recanalization with mechanical thrombectomy has brought about a paradigm shift in the optimal management of this high-risk group of patients, demonstrating significant benefits in clinical trials [2]. Importantly, early reperfusion is currently the only therapy that has proven to limit infarct size in patients with acute ischaemic stroke [1, 3]. However, a substantial number of those patients who receive treatment with tPA and/or thrombectomy in the acute phase do not ever fully recover [4, 5]. This reveals the need to develop new adjunctive neuroprotective treatment strategies alongside reperfusion therapy [6].

Although glucagon-like peptide-1 (GLP-1) therapy has been associated with the treatment of type 2 diabetes [7, 8], the ability of GLP-1 to activate pro-survival pathways is well known [9]. Importantly, a number of preclinical studies have demonstrated neuroprotective effects of GLP-1 in non-diabetic and diabetic models of acute ischaemic stroke [8, 10]. In total, all these studies suggest that the administration of GLP-1 receptor (GLP-1R) agonists is one of the most promising treatments to pursue for patients immediately after stroke.

The purpose of the current study is to compare the effects of two different GLP-1 analogues—liraglutide [11] and semaglutide [12–14]. Liraglutide is a first-generation GLP-1 analogue designed to have a reduced susceptibility to enzymatic degradation and an extended plasma half-life in humans of 13 h following subcutaneous administration, in comparison to the ~ 2-min half-life for human native GLP-1 [11]. Semaglutide is a newer GLP-1 analogue with a greatly prolonged half-life in humans of 165 h (approximately 1 week) [13, 14]. For this reason, we hypothesised that semaglutide would confer prolonged neuroprotection during the reperfusion period compared with shorter-acting GLP-1 analogues.

While neuroprotection by liraglutide has previously been demonstrated in a number of preclinical studies using permanent or transient middle cerebral artery occlusion (MCAO) [10], semaglutide has never been examined in either stroke model.

## Materials and Methods

### Animals and Experimental Groups

Male non-diabetic Sprague-Dawley rats (220–250 g) were used. All animals were randomly allocated to groups with allocation being concealed.

#### Study 1

The rats were subjected to i.v. bolus of vehicle (saline) (*n* = 6) or liraglutide via the tail vein at the onset of reperfusion following a 90-min MCAO. Three doses of liraglutide were used (350 μg/kg (*n* = 8), 700 μg/kg (*n* = 8) and 1050 μg/kg (*n* = 8)). These doses were selected based on a previously published study which showed that 700 μg/kg was neuroprotective in rats [15]. Functional status was evaluated 24 h after MCAO. The rats were then immediately sacrificed, and infarct sizes measured.

#### Study 2

The functional and infarct-limiting effects of the maximal dose of liraglutide from study 1 were evaluated in rats subjected to 90-min (*n* = 8), 120-min (*n* = 6) or 180-min (*n* = 5) MCAO, followed by 24-h reperfusion, in comparison with the corresponding time-matched control groups (*n* = 8, *n* = 8 and *n* = 5 at each time point).

#### Study 3

The functional and infarct-limiting effects of s.c. administration of liraglutide 1050 μg/kg (*n* = 9) and semaglutide 12 μg/kg (*n* = 10) 5 min before the onset of reperfusion were evaluated in rats subjected to 90-min MCAO, followed by 72-h reperfusion and compared with the control group (*n* = 8). The chosen dose of semaglutide was based on a previous publication [16]. To assess the role of GLP-1Rs in these effects, in one group of animals, the GLP1-R antagonist exendin(9-39) (Ex(9-39), 50 μg/kg, i.v.) was administered 15 min before the injection of semaglutide (*n* = 8). The dose of Ex(9-39) was selected on the basis of our previous study [17]. Blood glucose was measured in three random rats in each group to make sure no significant decline of glucose concentration occurs in response to the administration of these drugs.

### Transient Middle Cerebral Artery Occlusion

The intraluminal filament model of focal ischaemia was used [18, 19]. Briefly, under 2% isoflurane anaesthesia, a silicon-coated monofilament was advanced through the right common and internal carotid arteries towards the middle cerebral artery junction until resistance was felt (~ 2 cm). The animals were then allowed to recover in order to check for the presence of the functional signs of cortex brain ischaemia, such as walking towards the contralateral side, left forelimb flexion and body rotation to the left when held by the tail. Animals without any of these signs at this point were excluded from the study. The filament was withdrawn after 90, 120 or 180 min of occlusion.

### Functional Status Evaluation

Behavioural neurological evaluation was performed using three previously reported scoring scales or neuroscores [20–23]. These scales consist of simple sensorimotor tasks (spontaneous activity, gait, postural signs, lateral resistance, limb placing and parachute reflex) for the assessment of the severity of neurological deficits. Higher neuroscores reflect stronger deficits, with the maximal possible score being 22. The merged 0–22-point scale is presented in supporting information on the website.

### Infarct Size Measurement

The animals were euthanised with i.p. 100 mg/kg of sodium pentobarbital. The brains were immediately removed, sectioned at 1.25-mm intervals, stained with 1% triphenyl tetrazolium chloride (TTC) and fixed in formalin. The sections were photographed, and the resulting infarct areas were measured using ImageJ (the examples of the obtained images are presented on the website). Infarct sizes were presented as the hemispheric lesion volumes corrected for oedema (%HLVe) [24, 25].

### Statistical Analysis

One-way ANOVA (Dunn’s multiple comparison test) was used for statistical analysis of the data, following the Shapiro-Wilk normality test (GraphPad Prism 5, GraphPad Software, Inc., CA). Data are presented as median [25% percentile; 75% percentile]. Differences between groups were considered statistically significant when *P* < 0.05. Correlations were determined by Spearman’s *r* analysis.

## Results

### Liraglutide Reduces Brain Damage in a Dose-Dependent Manner

Study 1 investigated the potential neuroprotective efficacy of single bolus of either 350, 700 or 1050 μg/kg liraglutide administered at the onset of reperfusion.

**Fig. 1:**
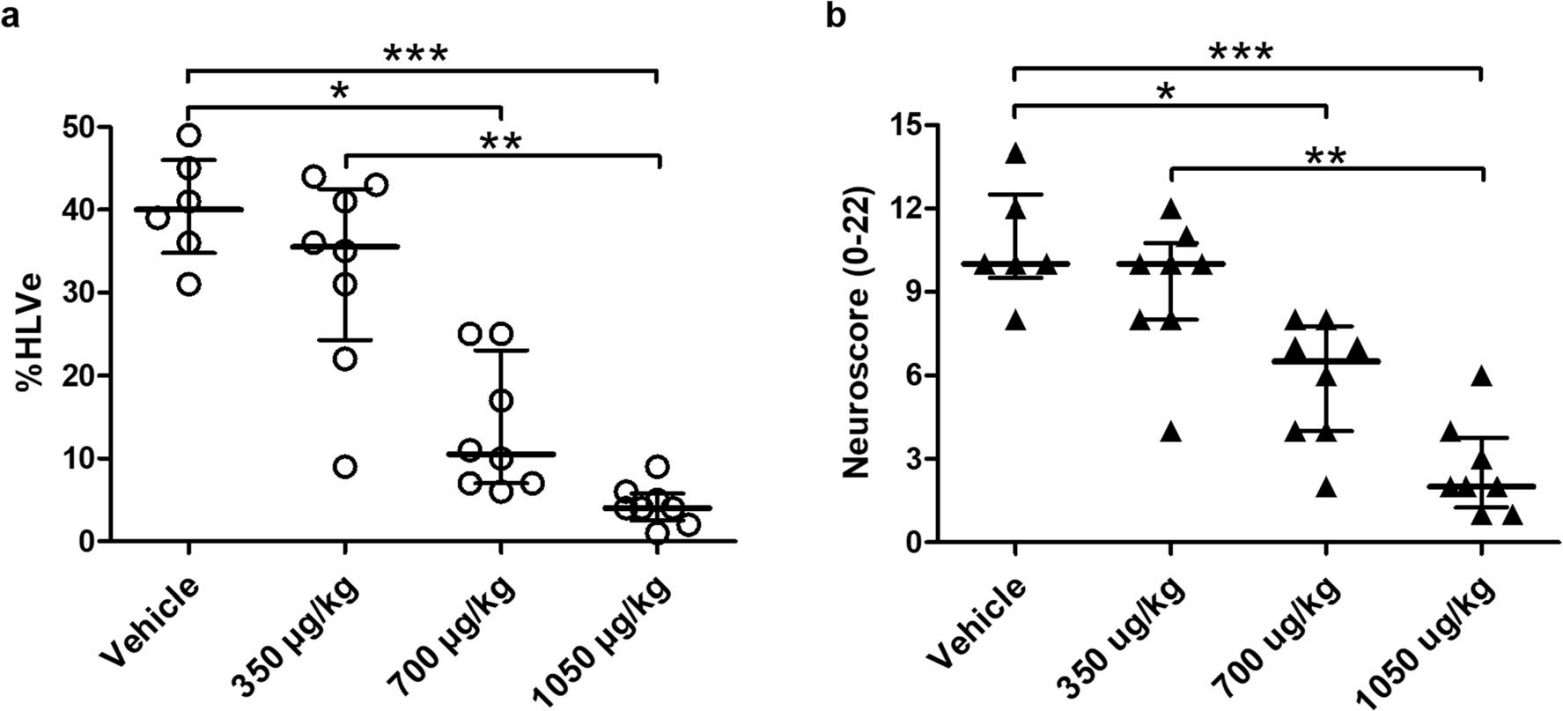
Neuroprotection induced by liraglutide administration in the acute ischaemic stroke model is dose-dependent. Rats were subjected to 90-min MCAO, followed by 24-h reperfusion. Vehicle or liraglutide was administered as i.v. bolus at the onset of reperfusion. Hemispheric lesion volumes corrected for oedema (%HLVe) (**a**) and neuroscores (**b**) were evaluated at the end of reperfusion period. *P < 0.05; **P < 0.01; ***P < 0.001

The %HLVe in the vehicle group was 40 [34; 46]. A total of 700 μg/kg or 1050 μg/kg liraglutide reduced infarct size by 74% (*P* < 0.05) and 90% (*P* < 0.001). No significant reduction in infarct size was observed in the animals treated with 350 μg/kg liraglutide (Fig. 1a). The median neuroscore after 24 h in the vehicle group was 10.0 (on the 0–22 scale) and was reduced with either 700 μg/kg (*P* < 0.05) or 1050 μg/kg (*P* < 0.001) liraglutide. No benefit was seen with 350 μg/kg liraglutide (median neuroscore of 10.0) (Fig. 1b). A significant correlation was found between infarct sizes and neuroscores on day 1 (*r* = 0.86; *P* < 0.001).

### Reperfusion Delay is a Limiting Factor for Neuroprotection by Liraglutide

In study 2, we investigated whether the neuroprotective efficacy of 1050 μg/kg liraglutide was retained when the duration of MCAO was extended to 120 and 180 min.

**Fig. 2:**
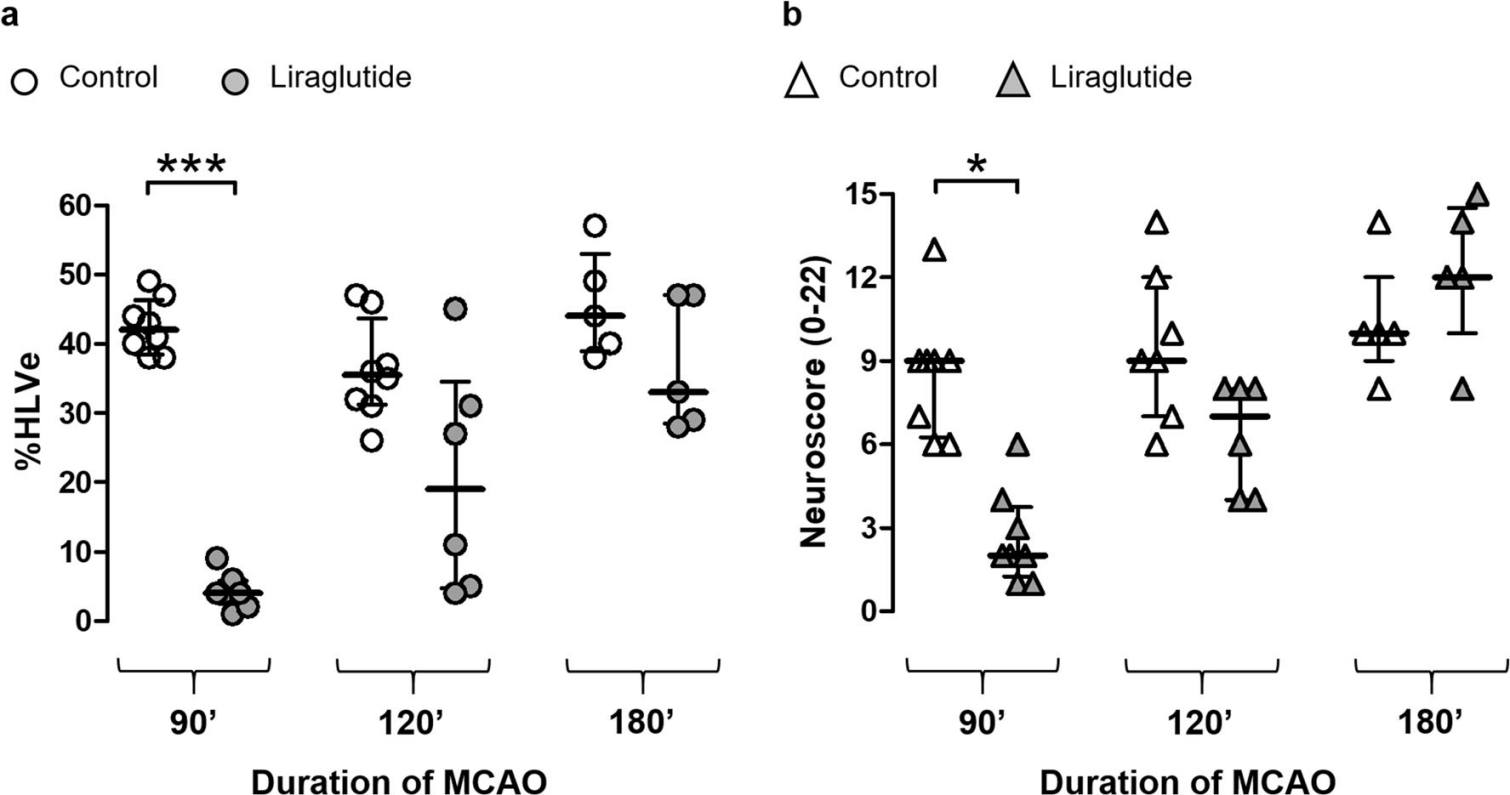
Reperfusion delay is a limiting factor for neuroprotection by liraglutide. Rats were subjected to 90-, 120- or 180-min MCAO, followed by 24-h reperfusion. Liraglutide was administered as i.v. bolus at the onset of reperfusion. Hemispheric lesion volumes corrected for oedema (%HLVe) (**a**) and neuroscores (**b**) were evaluated at the end of reperfusion period. Individual data and median with interquartile range are shown for each group. *P < 0.05; ***P < 0.001

The %HLVe in the control groups (MCAO duration of 90 min, 120 min and 180 min) were 42 [38; 46], 35 [31; 43] and 44 [39; 53] (*P* > 0.05 between all the control groups). Liraglutide reduced infarct size in the 90-min ischaemia groups (*P* < 0.001), but not in the 120- and 180-min groups (Fig. 2a). The median neuroscores in control groups subjected to 90-, 120- and 180-min ischaemia were 9.0, 9.0 and 10.0 respectively. In the corresponding liraglutide-treated groups, the medians were significantly reduced after 90-min (*P* < 0.05), but not 120-min and 180-min MCAO (Fig. 2b).

### Neuroprotection by Semaglutide Is at Least as Powerful as by Liraglutide and Is Mediated by GLP-1Rs

Increasing the duration of reperfusion period to 72 h in study 3 was accompanied by reduced survival: 2 out of 10 animals died in the control group, 2 out of 11 in the liraglutide group, and 3 out of 11 in the group in which the GLP-1R antagonist Ex(9-39) was administered before semaglutide. The only mortality-free group (0 out of 10) was that in which the rats were treated with semaglutide alone. However, none of these differences in mortality was significant.

In this series of experiments, visible intracerebral haemorrhage was observed in some brains at slicing: 5 from the control group, 1 from the liraglutide group and 2 from the Ex(9-39) + semaglutide group. No large visible haemorrhages were revealed in the rats treated with semaglutide without the GLP-1R antagonist.

**Fig. 3:**
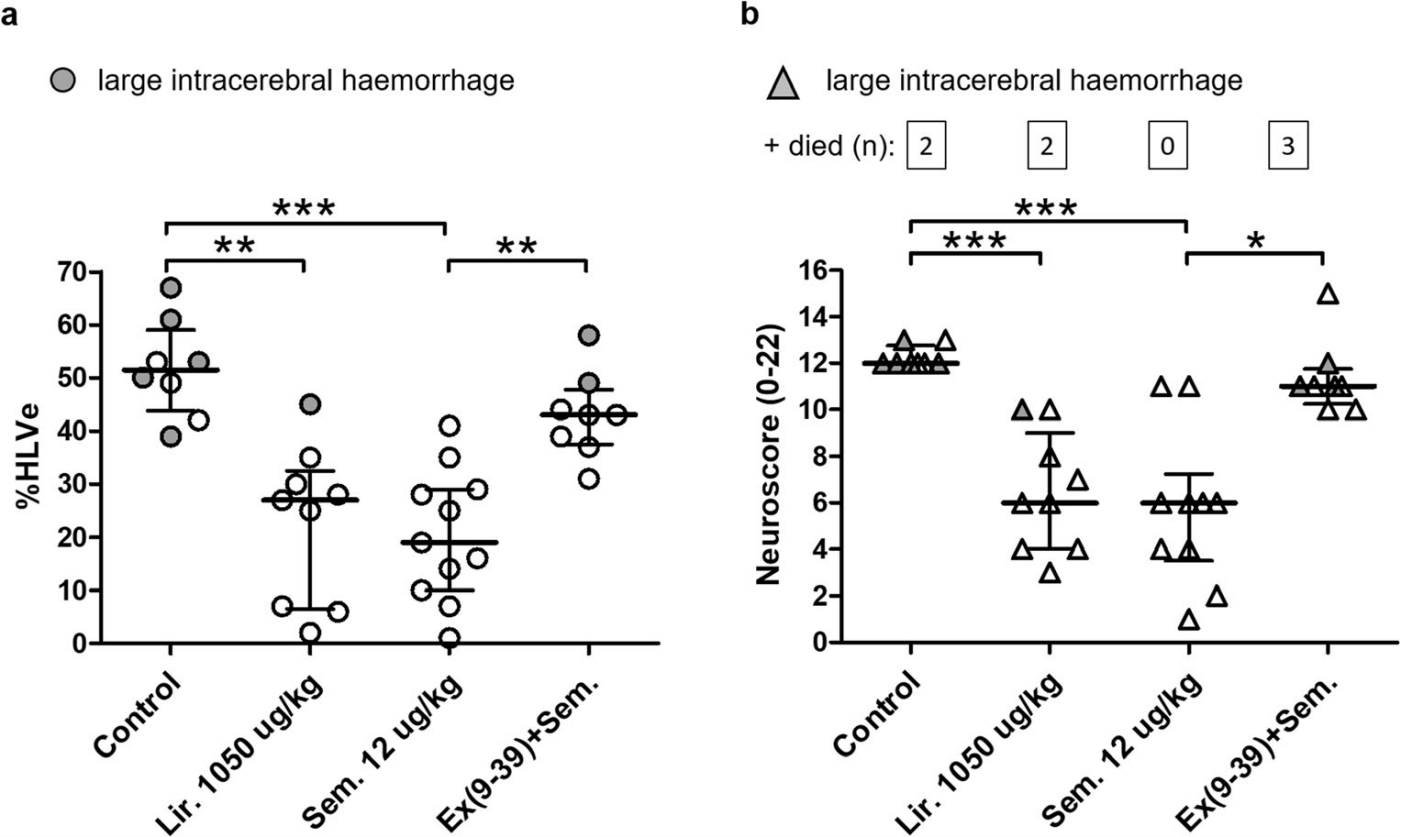
Neuroprotection by semaglutide is at least as strong as by liraglutide and is mediated by GLP-1Rs. Rats were subjected to 90-min MCAO, followed by 72-h reperfusion. Liraglutide (Lir.) or semaglutide (Sem.) was administered s.c. 5 min before the onset of reperfusion. Hemispheric lesion volumes corrected for oedema (%HLVe) (**a**) and neuroscores (**b**) were evaluated at the end of reperfusion period. Individual data and median with interquartile range are shown for each group. The numbers of the animals who did not survive 72-h reperfusion or had to be euthanised due to having reached the humane endpoints are presented in the boxes. *P < 0.05; **P < 0.01; ***P < 0.001

The %HLVe in surviving animals from the control group was 51 [43; 59]. Liraglutide and semaglutide each reduced infarct size at 72 h by 48% and 63% (*P* < 0.01 and *P* < 0.001 respectively). Ex(9-39) abolished the infarct-limiting effect of semaglutide (*P* > 0.05 vs. control) (Fig. 3a). The median neuroscore in the surviving animals from the control group was 12.0. In both liraglutide- and semaglutide-treated groups, the neuroscore was significantly reduced to 6.0 (*P* < 0.001). In rats treated with semaglutide and GLP-1R antagonist, the neuroscore was unchanged at 11.0 (Fig. 3b). A significant correlation was found between infarct sizes and neuroscores on day 3 (*r* = 0.9; *P* < 0.001).

## Discussion

Although the neuroprotective effects of liraglutide have been demonstrated previously in experimental studies [10], the current study is the first to show that the infarct-limiting effect of liraglutide is dose-dependent. Importantly, we used a clinically relevant model of transient focal brain ischaemia, as well as clinically applicable time of liraglutide administration, i.e., shortly before reperfusion. As the concentration and bioavailability of liraglutide can be variable following subcutaneous administration [14], we chose to administer it intravenously at the onset of reperfusion. According to data by Hunter and Hölscher, liraglutide is expected to cross the blood-brain barrier at the doses we tested, even in the normoxic state [26]. We regarded 1050 μg/kg as the maximal effective dose, as there were no significant differences in infarct size between 1050 and 700 μg/kg. We showed that the neuroprotective effects of liraglutide in an acute ischaemic stroke model are dose-dependent. The obtained data are in agreement with the study by Darsalia et al., demonstrating that the other synthetic form of GLP-1—exendin-4—reduces brain damage in a dose-dependent manner [27]. In addition, the infarct sizes in the current study correlated significantly with neuroscores, as in the study by Chauveau et al. [23].

It was shown previously that liraglutide administered 1 mg/kg per day does not reduce blood glucose level in non-diabetic rats [28]. Moreover, the neuroprotective effect of liraglutide in this study was independent of glycaemia normalisation [27]. For these reasons, we did not measure blood glucose dynamics in response to liraglutide treatment in all the animals, but only validated these previous data in three random rats of each group.

As the duration of brain ischaemia is a variable parameter in patients with an acute ischaemic stroke [29], in the second series of experiments, we examined the neuroprotective effects of 1050 μg/kg liraglutide bolus, administered at reperfusion, with more prolonged MCAO, specifically 120 and 180 min. Although there were no differences in infarct sizes between the control groups subjected to 90-, 120- and 180-min ischaemia, the infarct-limiting effect was not observed with 120-min and 180-min ischaemia. Similarly, the neuroscore improved significantly only in the 90-min treatment group. We were not able to find any direct evidence in the existing literature, as to the relationship between the duration of MCAO in rats and the corresponding ischaemic period in humans. However, it has been shown that rat brain infarcts increase in size progressively up to 120–180 min of MCAO [30], whereas reperfusion therapy in most patients with acute ischaemic stroke is effective within 6 h of focal brain ischaemia [3], suggesting ~ 2–3 times faster infarct progression in the brain of rats vs. humans.

In the third series of experiments, we compared the neuroprotective effects of liraglutide and the newer longer acting GLP-1 analogue, semaglutide. The principal clinic-related difference of semaglutide from liraglutide is the substantially longer half-life [11, 13, 14]. This means that the neuroprotection after single s.c. administration of semaglutide shortly before reperfusion could potentially cover a longer duration of the reperfusion process. For this reason, we extended the reperfusion period to 72 h to be able to observe the potential benefits of semaglutide due to its prolonged half-life. We showed that both these GLP-1 analogues reduce infarct size. Notably, the group treated with semaglutide was the only one in this series, where all the animals survived 72 h, and no large visible intracerebral haemorrhages were found. Although this latter observation still needs to be confirmed by further studies, this possible characteristic of semaglutide can be valuable for the patients with acute ischaemic stroke, especially those undergoing thrombolytic therapy [31–33]. Previously, it had been demonstrated that GLP-1R agonist exendin-4 ameliorates warfarin-associated haemorrhagic transformation after cerebral ischaemia [34]. Importantly, semaglutide has recently been shown in the SUSTAIN-6 trial to have clinical benefit in terms of reducing the rate of non-fatal stroke [34], although studies assessing functional outcome after stroke are still needed [35]. In this regard, our experimental study demonstrated improved functional recovery in rats treated with either liraglutide or semaglutide.

GLP1-R antagonist exendin(9-39) in our study abolished the neuroprotective effects of semaglutide, which indicates the key role of GLP-1Rs in these effects. This is in line with the previous study by Darsalia et al., where exendin-4 mediated neuroprotection in wild type, but not in *Glp-1r*-/- mice [36]. In addition, 2 animals in this group had large intracerebral haemorrhages. While GLP-1Rs are widely distributed in the brain [37, 38], the location of these receptors responsible for the neuroprotective effects is not known. However, regarding the possible protection from haemorrhage transformation of ischaemic stroke, the GLP-1Rs expressed in the endothelium [39] might be of more importance.

## Conclusion

This study demonstrates that the GLP-1 analogues, liraglutide and semaglutide, reduce infarct size in a model of acute ischaemic stroke in non-diabetic rats. We believe this study indicates the potential for agents to be used in the clinical setting of ischaemic stroke.

## Electronic Supplementary Material

ESM 1 (DOCX 1.88 MB)
