# Supplementary material for: Neuroprotection in Rats Following Ischaemia-Reperfusion Injury by GLP-1 Analogues—Liraglutide and Semaglutide
Source: Cardiovasc Drugs Ther. 2019 Nov 13;33(6):661–7. doi: 10.1007/s10557-019-06915-8 (PMC6994526; doi:10.1007/s10557-019-06915-8)
Supplement: Supplementary file 1 — (DOCX 1.88 MB) [file 10557_2019_6915_MOESM1_ESM.docx]

**Article title: Neuroprotection in rats following ischaemia-reperfusion injury by GLP-1 analogues – liraglutide and semaglutide**

**Journal: Translational Stroke Research**

**Authors:**

Maryna V Basalay,

Sean M Davidson,

Derek M Yellon*

The Hatter Cardiovascular Institute,

University College London

67 Chenies Mews, London WC1E 6HX

Corresponding author: Prof Derek Yellon

[d.yellon@ucl.ac.uk](mailto:d.yellon@ucl.ac.uk)

Phone: 07770470004

The Hatter Cardiovascular Institute

University College London

67 Chenies Mews

London WC1E 6HX

United Kingdom

**Neuroscore 0-22**

| **Sign** | **Description** | **Score** |
| --- | --- | --- |
|  |  |  |
| **Motility,**  **spontanous activity** | Normal | 0 |
|  | Slightly reduced exploratory behaviour | 1 |
|  | Moving limbs without proceeding | 2 |
|  | Moving only to stimuli | 3 |
|  | Unresponsive to stimuli, normal muscle tone | 4 |
|  | Premortal signs, severe hypotonia | 5 |
|  |  |  |
| **Gait** | Straight walking | 0 |
|  | Walking toward controlateral side | 1 |
|  | Alternate circling & straight walk | 2 |
|  | Alternate circling & walking toward paretic side | 3 |
|  | Circling/other gait disturbances | 4 |
|  | Constant circling toward paretic side | 5 |
|  |  |  |
| **Postural signs** | Degree of forelimb flexion when held by tail | 0-2 |
|  | Degree of body rotation when held by tail | 0-2 |
|  |  |  |
| **Parachute reflex** | Symmetrical | 0 |
|  | Asymmetrical | 1 |
|  | Controlateral forelimb retracted | 2 |
|  |  |  |
| **Lateral resistance** | Degree of resistance against lateral push | 0-2 |
|  |  |  |
| **Limb placing** | Ipsilateral forelimb: normal, weak, no placing | 0-2 |
|  | Controlateral forelimb: normal, weak, no placing | 0-2 |
|  |  |  |
| **TOTAL** |  | **0-22** |

**Supplementary Table 1:** Characteristics used in assessing the Neuroscore

| **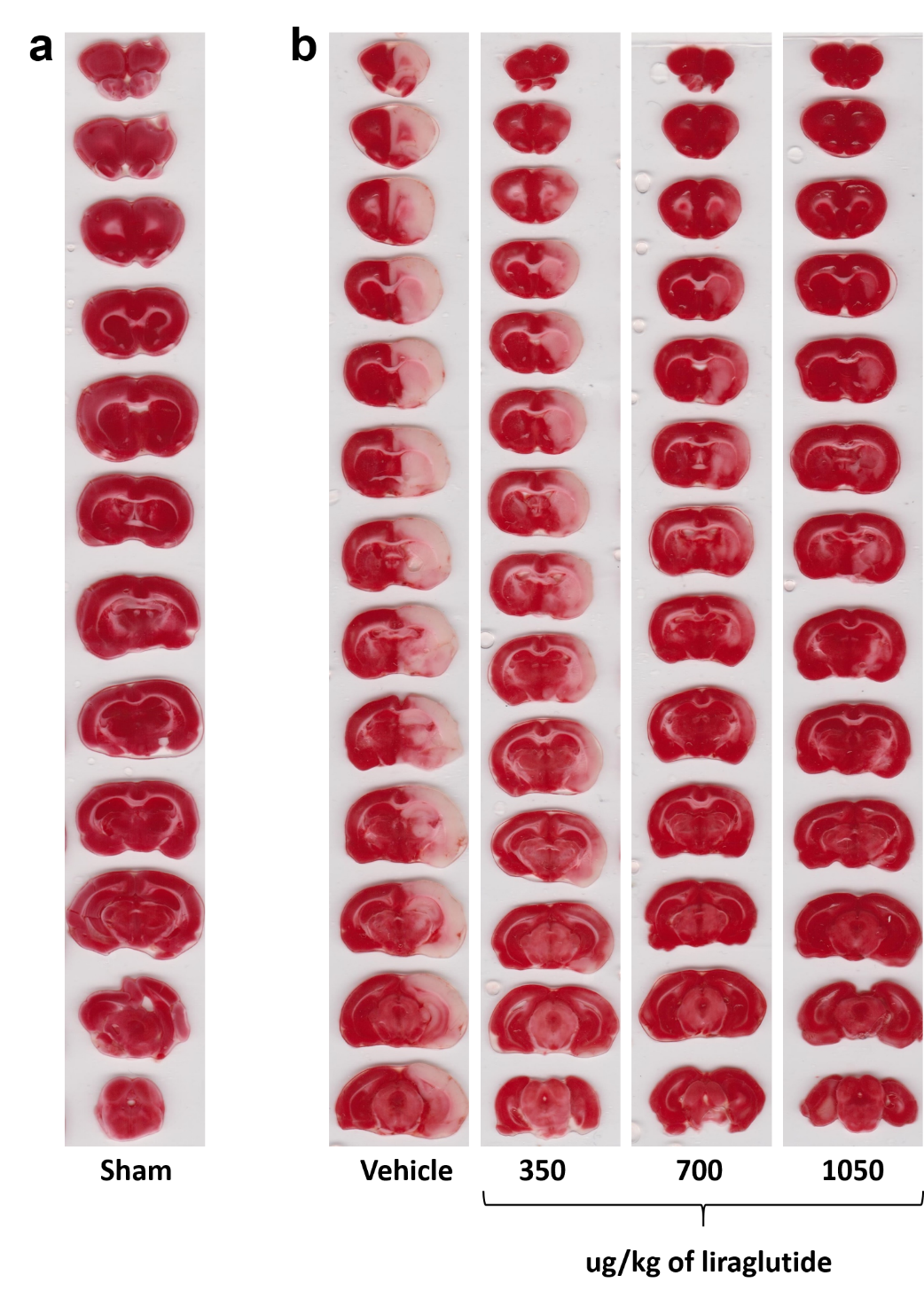** |  |
| --- | --- |

**Supplementary Figure 1:** The examples of the images of brain slices after TTC staining: each column of slices represents one brain of a rat after sham surgery (a) or included in Study 1 (b).
